# Parent and medical team disagreements in the UK: universal lessons in the origins and resolution in conflict

**DOI:** 10.1186/s44158-022-00075-2

**Published:** 2022-11-04

**Authors:** Karen Mackintosh, Paul McConnell

**Affiliations:** Anaesthetic Department, Royal Alexandra Hospital, Corsebar Road, Paisley, Scotland PA2 9PN

**Keywords:** Parental rights, Healthcare conflict, Children’s Act 1989, Conflict management frameworks

## Abstract

In recent decades, there has been an increase in life expectancy in children with life-limiting conditions. Ideally, parents and clinicians would work together to ensure the best care for these children. Several cases have appeared in the media in recent years where conflict has risen between parents and healthcare professionals acting in the ‘best interests’ of children, which have resulted in court action. However, the legislation itself promotes conflict. Similar laws exist across Europe based on Article 24 of the UN Convention on the Rights of the Child.

The aim of the Children’s Act 1989 in the UK was to place the ‘child’s welfare’ as the ‘paramount consideration’. It has prevented draconian care and supervision orders, which can only be made if the child is at risk of ‘significant harm’. This threshold does not apply to healthcare teams. Healthcare decisions are based on ‘best interests’, which are not explicitly defined. This sets the threshold for progression to court action much lower, and due to a lack of definitive definition of what ‘best interests’ are, this has unfortunately escalated conflict rather than resolve it.

Healthcare institutions have been criticised for not utilising alternative approaches first, such as mediation. We propose an alternative approach based on collaboration, reasonableness and the threshold of significant harm, which we have explored in this review.

Conflict management frameworks are a tool that can be used to recognise early signs of conflict and develop strategies to prevent escalation at ward level. They can be tailored to individual institutions and utilise content-oriented and empathetic communication strategies through designated clinicians. They should offer guidance on when to refer to the courts.

Parental wishes should be assessed on whether they represent significant harm or not. If not, they cannot simply be wrong. Acknowledgement of the ‘reasonableness’ of parental requests can be a key factor which is diffusing conflict. Therefore, setting the threshold for state intervention at ‘significant harm’ rather than ‘best interests’ would help to reduce the number of these cases progressing to courts.

## Background

In a perfect world, parents and healthcare teams would work in partnership to ensure the best care for seriously ill children [1]. Several high-profile cases have appeared in the media in recent years, where conflict has arisen between parents and healthcare professionals acting in the ‘best interests’ of children leading them to seek resolution in court [2–4]. Criticisms have been directed at all invested parties in these cases; however, the legislation itself is at fault in producing this conflict. In this review, we will examine the Children’s Act in the UK and its role in promoting conflict within healthcare. This will allow us to examine universal alternative approaches applicable across jurisdictions, which are more suited to deal with this emotive area of law.

## The Children’s Act

The purpose of the Children’s Act 1989 in the UK was to reform the law relating to children with the emphasis on ‘the child’s welfare’ being ‘the paramount consideration’ [5, 6]. Similar laws exist across Europe ground in Article 24 of the UN Convention on the Rights of the Child that the child is paramount [7]. It defines the role of parents and the power and actions available to the court. Any decision made should be in the child’s ‘best interests’, which (unfortunately) the Act does not explicitly define [8]. Section 1 (5) advises the court should only use its extensive powers, if it considers doing so would be better than making no order at all [9]. This was endorsed as providing ‘a practical answer to a practical problem’ giving ‘direction for the purpose of determining a specific question which has arisen or may arise’ [10, 11]. Any individual with a ‘close interest’ to a child can apply to the courts, which, as Herring discusses, seeks ‘to strike a balance between making the court accessible to all those who have legitimate concerns and protecting those who are, from the stress of facing challenges to their parenting in the courts’. Decisions are based on ‘best interests’ test [12].

Care and supervision orders can only be made if child is ‘suffering or likely to suffer significant harm’ [13]. While this goal of the Act is to prevent draconian care orders and limit the powers of local authorities, this does not apply to healthcare teams. Lady Hale clarified the distinction as follows: ‘…. the significant harm requirement does not apply to hospitals asking for guidance as to what treatment is and is not in a child’s best interests’ [14]. A hospital is therefore allowed to ask the courts at the low threshold of ‘best interests’ to intervene, and the courts are entitled to decree with the full scope of their powers. Several court cases have unfortunately shown the impact of the ‘best interests’ test, where rather than resolve conflict they have escalated it.

## Parental rights and healthcare

In a legal sense under UK law, parents do not have rights. They have obligations to their children, which allows them to fulfil their responsibilities in caring for them. In healthcare, this allows parents to consent to treatment by healthcare professionals on a child’s behalf. However, they ‘are not entitled to insist upon treatment by anyone which is not in their child’s best interests’ as stated by Lade Hale, diminishing the parental role and by extension their pain and anguish that comes with caring for an unwell child [14]. This creates a disparity between clinicians and parents setting the foundations for conflict to occur. Healthcare professional’s primary focus is acting in the child’s ‘best interests’ from the initial consultation through to diagnosis and treatment. ‘Best interests’ here though are heavily influenced by their medical bias.

There is ‘a lack of understanding of the responsibilities of professional and public authorities when a conflict arises about a child’s care’ and an unclear threshold when to progress to court involvement [14]. Upon reaching the courts however, disparity between medical staff and parents is even more pronounced. While parents are bound by all court decisions, courts cannot compel a doctor to act against their ‘professional conscience’ [15]. Clinical teams may apply to the courts to green light their medical decisions or offer a judicial absolution for their consciences, while the parents’ actions are not judged on ‘reasonableness’ or safety. They are the individuals who bear the long-term consequences and deal with the direct impact on their family [16]. Clinicians and courts move on to the next patient or case.

The Children’s Act sought to create a single statutory framework to reflect ‘a coherent set of legal concepts and principles’ [17]. This same legislation is applied to abuse, neglect and significant harm cases as well as healthcare disagreements between caring parents and clinicians, priming them for further conflict. Vague concepts and statements like ‘best interests’ and ‘welfare is paramount’ can lead to differing interpretations between invested parties causing distress, confusion and inconsistencies compounding inflammatory situations further. Once ‘best interests’ are invoked, there can only be one ‘best interest’, and that will be prescribed by the court. Parents who believe they are acting in the best interests of their child, coming from a place of love and beneficence, may be told they are wrong; they are not doing ‘the best’ for their child decreed via an Act more associated with negligence and neglect. This is further compounded by inconsistencies within UK case law, which often disempowers parents, who believe they are doing what is right for their child.

## UK case studies

In 1981 Re B, the court was asked to rule on whether lifesaving bowel surgery should be carried out on a baby with Down syndrome [18]. The treating medical team was in favour of surgery, which the parents opposed. In his summary, Dunn L. J. acknowledged having ‘great sympathy for the parents in the agonising decision to which they came…But the child now being a ward of the court, although … the decision of the parents, which …. was an entirely responsible one, doing what they considered was best, the fact of the matter is that this court now has to make the decision’ [18]. Though, there was recognition in the reasonableness of the parents’ position, ultimately, the court would rule solely based on the ‘best interests’ test. In future cases where the ‘best interests’ test and paramountcy were invoked, these concepts were interpreted very differently.

In Re King, the child had undergone surgical management for a medulloblastoma, which required further radiotherapy [19]. He was offered conventional radiotherapy; however, his parents wished for ‘proton beam therapy’ not available on the NHS. Although his clinical team had researched the intervention, an NHS commissioning team declined it as a treatment option. It was felt the additional benefits were voided by the extent of the radiotherapy required. The child’s parents removed him from the hospital; concerned about the risk of harm to the patient, courts were notified. An arrest warrant was issued for the parents. The parents were able to demonstrate they had sourced an alternative treatment plan and were worried having disagreed with the medical team, their child would be taken into care. Fundamentally, there was a breakdown in communication between both parties. The health board was reprimanded for this: ‘Disputes over best interests will be not be settled with arrests’ [20]. Baker J. summarised the following: ‘In most cases, the parents will the best people to make decisions about a child and the state – whether it be the courts or any public authority – has no business interfering with the exercise of parental authority unless the child is suffering or is likely to suffer significant harm…’ [19]. He later announced that where both treatment options were reasonable, ‘…it is the parents who bear responsibility of making the decision. It is no business of this court or any other public authority to intervene’ [19]. Introducing ‘reasonableness’ into the decision-making process, disguised as ‘best interests’, further illustrates confusion in current law.

The case of Charlie Gard highlighted further inadequacies in the subjective use of ‘best interests’. His parents were denied leave to seek an experimental treatment in the USA as it was deemed to have little chance of success. It was in his ‘best interests’ to discontinue his supportive treatment as per the medical team. Birchley pointed out the harm of ongoing ventilation was static; rather, the benefit of it had diminished. If a treatment option still existed, then the benefit would outweigh the harm [21]. The parents had found another medical team willing to treat him rather than asking his current doctors to act against their clinical judgement. By placing ‘best interest’ as the standard and trigger for intervention, this creates a situation which generates conflict and distress as evidenced in this case.

Whilst ruling in another case, Hewson comments that least destructive forms of dispute resolution should have been deployed first [22]. Highlighted by these case studies, it is a failing of our current system that alternative methods and reconciliation are not exhausted first.

## Alternative approach — collaboration, reasonableness and significant harm

Factors that can contribute towards conflicts between parents and clinicians include poor communication, uncertainty regarding patient diagnosis or prognosis, strong negative emotions like anger and limited health literacy and high burden of responsibility for decision-making [23].

Good communication and working in collaboration with parents can help to reduce conflict. It involves avoiding giving unrealistic expectations, assigning a lead clinician to liaise with families to deliver clear and consistent messaging, addressing concerns and involving palliative care teams early for symptom management and providing psychological support to families and staff [24].

If conflict does arise, identify it early! Early signs can include clinicians and parents avoiding each other or not engaging when communication is attempted. Families may attempt to micromanage everything or play professionals off against each other. Recognising these signs can prompt early intervention to allow parents to express their concerns. Healthcare institutions should utilise mediation and conflict management frameworks to reduce conflict and reduce staff burnout [25].

Conflict management frameworks are a tool, which can be tailored to individual institutions with the aim to resolve difficulties at ward level. They allow identification of triggers implying conflict and utilisation of communication plans and huddles, ward champions and designated clinicians to reduce this. They should include an escalation plan including guidance on when to refer to management, child protection services and the courts.

Communication strategies like content-orientated or empathetic ones were found to be effective in preventing conflicts from escalating. Content-oriented strategies are effective in managing conflict with regard to one topic. These include acknowledging opposing views on treatment, clarifying by providing factual information or reformulating such as reiterating what the medical team has previously said. In more complicated conflicts, an empathetic approach was found to be more useful by acknowledging emotional situations, encouraging families to share their views and providing emotional support [23].

In the UK, setting the threshold for court engagement at ‘best interests’ rather than ‘significant harm’, progression to the courts can rapidly escalate and exacerbate conflict. Through careful communication and utilising conflict management frameworks, clinicians should assess if parents’ wishes represent significant harm or not. Amongst ethicists, there is substantial consensus harm should be the central moral concept when judging the appropriate threshold for state intervention [26]. If there is no immediate significant harm, mediation should be employed first rather than recourse to the courts.

## Conclusions

The number of children living with complex and life-limiting conditions is rising as is the opportunity for parent/healthcare conflict. To identify a single best course of action is a fallacy as it is impossible to have all the information. The child themselves is often ‘beyond experience’, and our common approach of substituted judgement is unsuitable [27]. ‘If there can be reasonable disagreement, then the parental view cannot (simply) be wrong’ [28]. There is scope for different views, and a key factor in diffusing or preventing conflict is acknowledging the ‘reasonableness’ of parental requests. Good consistent communication is key, and should it be insufficient, conflict management frameworks and mediation can help avoid progression to the courts.

## Data Availability

Not applicable
